# A Non-Surgical Treatise on the Diseases of the Prostate Gland

**Published:** 1906-09

**Authors:** 


					﻿A Non-Surgical Treatise on the Diseases oe the Pros-
tate Gland and Adnexa. By George W. Overall, A.B.,
B.D., Chicago. Rowe Publishing Co.
The exhaustion in less than a year of the second double edition
of this book shows the favor with which the work of Dr. Overall
has been received. The title, however, is misleading, for although
it deals with the non-operative treatment, most of measures
recommended should be properly classed as surgical.
Among the additions to this edition is an endoscope for treat-
ing the prostatic urethra; a new electrode devised for applying
electricity to the prostate through the rectum, and an electric
cautery to be used through the systoscope.
As in the former editions, overmuch is given of electrical treat-
ment. Much valuable information, however, is contained in this
little book.
X-Ray Diagnosis of Kidney Stone.
Holland sums up his paper as follows: When a stone or stones
are present in such size as to produce symptoms suggesting the
desirability of operation, if a careful and thorough examination is
made such stone or stones can nearly always be shown by X-rays.
And this would also apply to the presence of stone even if the
symptoms alone were not sufficient to demand operation. In most
cases when shadows are shown the size, shape and position can
be relied on in diagnosing them as from kidney or ureteral stones.
In other cases when doubt may arise as to the cause of the shadows
the experience of the examiner will often settle the matter, and in
some cases stereoscopic radiography, the use of ureteral bougies,
etc., can be used as a help. The negative diagnosis can be relied
on only when the whole region on both sides is carefully examined
and when the plates are taken under the essential condition for
successful examination and when they are of the necessary quality
in showing sufficient differentiation of the soft structures in the
kidney and ureteral regions. There can be no justification for
operation or prolonged medical treatment without an efficient X-
ray examination being made.—Lancet, June 2, 1906.
In all cases of torticollis, examine for caries of the spine.—
American Journal of Surgery.
				

